# Theory‐Guided Low‐Speed Centrifugation for High‐Purity Boron Nitride Nanotubes

**DOI:** 10.1002/smsc.70359

**Published:** 2026-07-31

**Authors:** Thang Quoc Huynh, Jun‐Hyung Im, Eunsu Cho, Jin‐Kang Choi, Kwang‐Suk Oh, Young‐Ki Kim

**Affiliations:** ^1^ Department of Chemical Engineering Pohang University of Science and Technology (POSTECH) Pohang Republic of Korea

**Keywords:** boron nitride nanotubes, high purity, liquid crystal, low‐speed centrifugation, purification

## Abstract

Boron nitride nanotubes (BNNTs) are promising building blocks for next‐generation technologies, yet synthesis‐derived impurities and structural damage from conventional purification methods (e.g., harsh acid treatments, high‐speed centrifugation) hinder their practical applications. Here, we report a theory‐guided low‐speed centrifugation strategy for high‐purity BNNT enrichment without detectable structural damage. Leveraging fundamental sedimentation principles, this approach capitalizes on the distinct sedimentation contrast between compact impurities and high‐aspect‐ratio BNNTs. This mechanism enables highly selective separation, where impurities sediment rapidly while BNNTs remain suspended. Comprehensive experimental validations confirm that estimated BNNT purity approaching ∼95% is achievable, providing a favorable purity–recovery balance while preserving nanotube morphology and dispersibility under the tested conditions. The strategy exhibits versatility across tested surfactant systems in environmentally benign aqueous media, eliminating the need for strong acids or organic solvents while maintaining compatibility with ionic and nonionic stabilizers. Importantly, the purified BNNT dispersions retain their capacity for Onsager‐type anisotropic assembly to form uniformly aligned architectures during subsequent gravity filtration. This offers direct opportunities for advanced anisotropic applications, including thermal management, mechanical reinforcement, and directional transport. By unifying sedimentation theory with practical processing, this work establishes low‐speed centrifugation as a viable and sustainable strategy for high‐aspect‐ratio one‐dimensional nanomaterials.

## Graphical abstract

This study presents a theory‐guided, low‐speed centrifugation strategy for boron nitride nanotube (BNNT) purification, compatible with various surfactant systems. The method selectively removes impurities while preserving nanotube integrity and length. Combined theoretical analysis and experimental validation confirm high estimated purity and structural stability, demonstrating a mild, proof‐of‐concept approach to facilitate the anisotropic assembly of pristine BNNTs for advanced material applications.

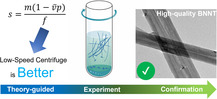

## 1 Introduction

Boron nitride nanotubes (BNNTs) have garnered significant attention in nanomaterials science due to their remarkable properties—including high thermal conductivity, exceptional mechanical strength, chemical stability, and electrical insulating behavior—which rival or surpass those of carbon nanotubes (CNTs) in specific applications [1, 2, 3]. These attributes position BNNTs as a critical component in next‐generation technologies, ranging from electronics to biomedical applications, such as high‐performance polymer composites, thermal management systems, and biocompatible scaffolds [2]. However, existing synthesis methods for BNNTs (e.g., chemical vapor deposition, laser ablation, or high‐pressure high‐temperature processes) often result in heterogeneous products contaminated with impurities, including amorphous boron, hexagonal boron nitride (h‐BN), and residual metal catalysts [4, 5, 6]. These impurities severely compromise the performance and reliability of BNNTs in practical applications, necessitating robust purification strategies to achieve high‐purity BNNTs while preserving their structural integrity.

For BNNT purifications, three representative methods—i) acid treatment, ii) solvent dispersion, and iii) high‐speed centrifugation—have been widely utilized, yet significant limitations remain. Specifically, acid treatments, often employing strong acids like chlorosulfonic acid, effectively dissolve metal catalysts and boron oxides but risk damaging the BNNT structures through chemical etching, leading to tube shortening or surface defects [7, 8]. Another approach is the solvent‐dispersion method, in which BNNTs are directly dispersed in liquids (e.g., water or alcohol) under ultrasonication or hydrothermal treatments in an autoclave. The resulting suspension is subsequently allowed to stabilize under mild conditions, enabling impurities to gradually separate from the BNNTs through differential sedimentation. Compared to acid treatments, this method offers a gentler approach that reduces the structural damage to BNNTs; however, it often struggles to remove impurities efficiently [9, 10]. Furthermore, these two methods are limited in terms of practical processing because they generate chemical waste (e.g., toxic solvents and acids) or require the energy‐intensive heating of entire solutions for extended periods, raising both environmental and economic concerns.

Lastly, the centrifugation‐based purification method, which leverages density gradients to separate BNNTs from heavier impurities such as h‐BN, has been considered a standard approach due to its minimal chemical risk. However, while capable of achieving purities of up to 90% [11], conventional centrifugation strategies predominantly rely on high‐speed centrifugation with rotor speed above 10,000 rpm. This reliance results in critical limitations: The excessive centrifugal force often fails to distinguish BNNTs from similarly dense h‐BN platelets, leading to cosedimentation, low yields, and poor selectivity. Furthermore, the dependence on costly ultracentrifuges to achieve such speeds (≥10,000 rpm) limits practical throughput, and the accompanying intensive mechanical stresses can cause tube bending or wall collapse, thereby compromising BNNT structural integrity [3, 9]. To address these challenges, low‐speed centrifugation has been noted in prior studies, but it has yet to be systematically investigated, leaving its separation mechanism elusive and its potential largely overlooked. Existing reports failed to explain how mild centrifugal forces can selectively remove impurities while retaining BNNTs in suspension, fostering misconceptions regarding the effectiveness of low‐speed centrifugation methods [3, 12, 13].

Here, we present a theory‐to‐experiment study demonstrating that low‐speed centrifugation at 3000 rpm provides an effective and fundamentally distinct route for BNNT purification. Guided by the Svedberg equation and Stokes’ hydrodynamic drag law, this approach exploits the pronounced contrast in sedimentation behavior between compact, near‐spherical impurities and high‐aspect‐ratio BNNTs, enabling the efficient removal of amorphous boron and h‐BN while maintaining BNNTs in suspension. As a result, an estimated BNNT purity of 94.7% ± 1.5% is obtained without detectable structural damage or loss of nanotube length. The resulting high‐purity BNNT dispersions further exhibit an enhanced capacity for Onsager‐type anisotropic assembly during gravity filtration, enabling the formation of uniformly aligned nanotube assemblies for advanced anisotropic applications. Beyond its selectivity and gentleness, the process operates in environmentally benign media, maintains compatibility with a broad range of surfactant systems, and shows promising scalability. By integrating analytical predictions with comprehensive experimental validation, this work establishes low‐speed centrifugation as a viable and sustainable processing strategy for high‐purity BNNT enrichment and assembly.

## 2 Results and Discussion

### 2.1 Theoretical Analysis of Low‐Speed Centrifugation Purification

**FIGURE 1:**
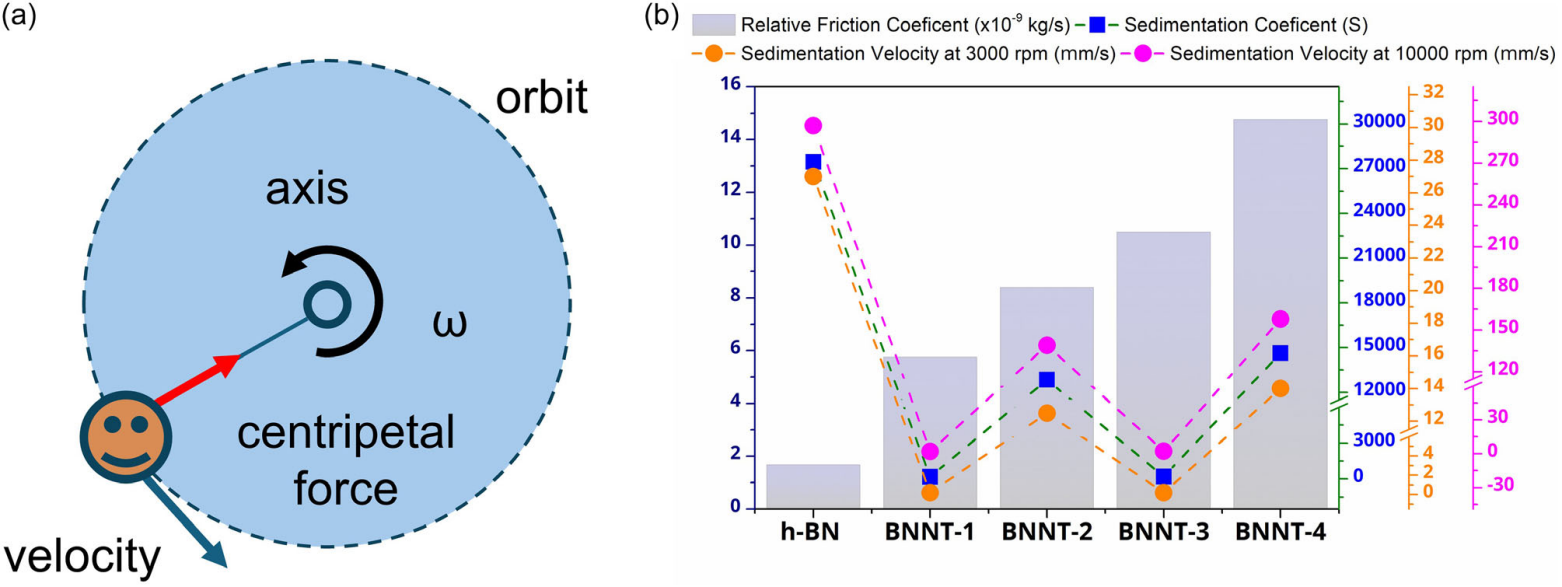
(a) Simplified centrifuge working principle. (b) Calculated relative friction coefficient (*f*, gray bars), sedimentation coefficient (*s*, blue squares), sedimentation velocities (*v*) at 3000 rpm (orange circles), and 10,000 rpm (pink circles). In this theoretical analysis, we assume the h‐BN impurity as a quasispherical particle with a radius of *r*_p_ = 100 nm and the BNNTs as cylindrical particles with length and diameter as follows: BNNT‐1: 5 μm × 5 nm; BNNT‐2: 5 μm × 50 nm; BNNT‐3: 10 μm × 5 nm; BNNT‐4: 10 μm × 50 nm.

Centrifugation is a fundamental laboratory technique used to separate substances based on their density by spinning them at high speeds (Figure 1a). During this process, particles rotate around the centrifugal axis with an angular velocity (*ω*) and experience a centrifugal force that drives radial motion through the fluid. The resulting particle velocity is determined by the balance between this outward force and hydrodynamic drag, causing particles with different effective masses and hydrodynamic resistances to migrate away from the rotation axis at distinct rates [14, 15]. To enable the practical and nondestructive purification of BNNTs, we establish a low‐speed centrifugation strategy guided by the particle sedimentation velocity and hydrodynamic drag of BNNTs in water. In this process, the nonionic surfactant of Tween 80 is incorporated into the aqueous BNNT solutions, playing an important role in dispersing the BNNTs individually and preventing aggregation, thereby allowing for the selective separation of impurities (further discussed in Figure 4). BNNTs, defined by a high‐aspect‐ratio tubular morphology, contrast sharply with common synthesis byproducts, such as metal catalysts and h‐BN, which can be approximated as quasispherical particles. Therefore, the selectivity of the low‐speed centrifugation protocol can be qualitatively explained by combining the Svedberg equation with Stokes’ hydrodynamic drag law, expressed as:

$$s=\frac{m\left(1-\bar{v}\rho_{0}\right)}{f}$$

where *s* is the sedimentation coefficient, *m* is the particle mass, $\bar{v}$ is the partial specific volume of the particle (for as‐received BNNTs containing impurities, $\bar{v}$ ≈ 4.762 × 10^−4^ m^3^/kg with the particle density of *ρ*_p_ = 2100 kg/m^3^) [16], *ρ*_0_ is the density of the solvent (water, *ρ*_0_ = 1000 kg/m^3^), and *f* is the frictional coefficient of the particle that governs its sedimentation behavior.

Specifically, using a 50 mL conical tube (length: 0.115 m) and a centrifuge with a rotor radius (*r*_r_) of 0.0985 m, we analyze the sedimentation coefficient (*s*) and sedimentation velocities (*v*) for h‐BN as a representative impurity (radius *r*_p_ = 100 nm) and various types of BNNTs, all of which are dispersed in water at 25°C. The BNNTs utilized in this analysis consist of four representative types: i) BNNT‐1 (length *L* = 5 μm × diameter *D* = 5 nm), ii) BNNT‐2 (5 μm × 50 nm), iii) BNNT‐3 (10 μm × 5 nm), and iv) BNNT‐4 (10 μm × 50 nm). These dimensions reflect typical BNNTs synthesized via a ball‐milling method employed in this work, as well as those produced by other established synthetic routes [6]. We note that this sedimentation model is intended as a simplified qualitative framework rather than a fully predictive description. The h‐BN impurities are approximated as compact, quasispherical particles to contrast their behavior with that of the high‐aspect‐ratio BNNTs. A 100‐nm impurity is chosen as a conservative representative case, because larger h‐BN particles and aggregates sediment more readily due to their higher effective mass and sedimentation velocities [11]. Therefore, the calculated velocities are used to rationalize the observed separation trend rather than to provide exact separation‐time predictions.

For the spherical h‐BN impurity, the frictional coefficient *f* (gray bar in Figure 1b) is calculated via Stokes’ law as *f* = 6π·*η*·*r*_p_ = 1.678 × 10^−9^ kg/s (where the water viscosity *η* = 8.9 × 10^−4^ Pa·s), yielding a high sedimentation coefficient of *s* = 2.747 × 10^4^ S (blue square in Figure 1b). In contrast, for the cylindrical BNNTs, the Broersma–Tirado approximation [17, 18], *f* = 3π·*η·L*/[ln(2*L*/*D*) – 0.307], which accounts for the high‐aspect‐ratio geometry of BNNTs, results in a higher *f* value (5.750 × 10^−9^ to 14.76 × 10^−9^ kg/s, gray bars in Figure 1b) and a lower *s* value (2.057 × 10^2^ to 1.464 × 10^4^ S, blue squares in Figure 1b). These differences between h‐BN and BNNTs stem from the greater hydrodynamic drag experienced by BNNTs due to their elongated shape and, particularly for thinner BNNTs (i.e., BNNT‐1 and BNNT‐3 with *D* = 5 nm), their substantially lower masses (e.g., *m* = 4.124 × 10^−19^ kg for BNNT‐3 vs. *m* = 8.796 × 10^−18^ kg for h‐BN). Detailed calculations are elaborated in Supporting Information (Part 1).

In terms of the sedimentation velocity *v*, we also find h‐BN and BNNTs to be markedly differentiated. At 3000 rpm (corresponding to *ω* ≈ 3.142 × 10^2^ rad/s), we calculate that the h‐BN sediments 1.88–146 times faster than the BNNTs, with the most pronounced disparities observed for the thinner BNNT‐1 and BNNT‐3 (orange circles in Figure 1b). Specifically, the calculated sedimentation velocities are *v* = 27 μm/s for h‐BN, 0.18 μm/s for BNNT‐1, 12.5 μm/s for BNNT‐2, 0.2 μm/s for BNNT‐3, and 14 μm/s for BNNT‐4. During a 10‐min centrifugation in the 0.115‐m‐long centrifuge tube, therefore, h‐BN travels approximately 0.0160 m (13.9% of the tube length), whereas the BNNTs travel significantly less: BNNT‐4 travels 0.0085 m (7.4%), and BNNT‐3 travels only 0.00012 m (0.10%). Based on the predicted values of *v*, the sedimentation times for traversing the full tube length of 0.115 m (*t*_full_) are calculated to be approximately 71.81 min for h‐BN, 174.98 h for BNNT‐1, 153.46 min for BNNT‐2, 159.72 h for BNNT‐3, and 134.79 min for BNNT‐4. This significant temporal separation ensures that h‐BN impurities reach the pellet within a typical 10–30 min centrifugation run under practical experimental conditions, where the centrifuge conical tube is tilted; this tilting shortens the effective sedimentation path length and results in faster settling of impurities than the prediction of the idealized theoretical model (*t*_full_ = 71.81 min for h‐BN). However, the BNNTs, even those with larger diameters (i.e., BNNT‐2 and BNNT‐4) that exhibit much shorter *t*_full_ than thinner ones (i.e., BNNT‐1 and BNNT‐3), can still remain suspended in the supernatant, facilitating high‐purity separation. We note that the conical tube geometry, narrowing toward the bottom, also reduces the effective sedimentation distance for particles near the pellet, but the 0.115‐m length ensures robust separation at low speed.

In contrast, at the higher speed of 10,000 rpm (corresponding to *ω* ≈ 1.047 × 10^3^ rad/s) used in the conventional high‐speed centrifugation methods, *v* increases significantly for both h‐BN and BNNTs (pink circle in Figure 1b); *v* = 297 μm/s (*t*_full_ = 6.46 min) for h‐BN, *v* = 2 μm/s (*t*_full_ = 15.99 h) for BNNT‐1, *v* = 139 μm/s (*t*_full_ = 13.80 min) for BNNT‐2, *v* = 2.2 μm/s (*t*_full_ = 14.54 h) for BNNT‐3, and *v* = 158 μm/s (*t*_full_ = 12.13 min) for BNNT‐4. Because the particles reach the pellet much faster than the theoretically estimated *t*_full_ under the practical experimental condition (due to the tilted centrifuge tube), this narrowed time window causes the thicker BNNTs (i.e., BNNT‐2 and BNNT‐4 with *D* = 50 nm) to cosediment into the pellet alongside h‐BN. This leads to a significant reduction in the yield of purified BNNTs recovered from the supernatant.

The effectiveness of low‐speed centrifugation at 3000 rpm lies in its ability to exploit the distinct sedimentation behaviors of particles to achieve both high purity and high yield. The reduced *v* leads to an extended sedimentation period for particles, providing a broader time frame for precise control over the centrifugation duration to ensure that h‐BN impurities reach the pellet while the majority of BNNTs remain in suspension. For instance, while h‐BN reaches full sedimentation in *t*_full_ = 71.81 min, the thinner BNNT‐1 and BNNT‐3 (*D* = 5 nm) require *t*_full_ = 174.98 and 159.72 h to sediment, respectively. This vast disparity in sedimentation time ensures that the BNNTs remain in the supernatant, even during extended centrifugation runs. This is particularly advantageous for BNNT samples with a heterogeneous size distribution, as the low‐speed centrifugation preserves a broader range of BNNTs in the supernatant, thereby maximizing the yield of the purified nanotubes. However, under the conventional high‐speed centrifugation (≥10,000 rpm), the rapid sedimentation of thicker BNNTs (*t*_full_ = 13.80 min for BNNT‐2 and *t*_full_ = 12.13 min for BNNT‐4) leads to a significant risk of cosedimentation with h‐BN (*t*_full_ = 6.46 min), resulting in reduced yield and compromised purity. Additionally, the low‐speed centrifugation minimizes the risk of BNNT aggregation because higher centrifugal forces promote increased interactions between particles via Van der Waals forces, a challenge analogous to that observed at high volume fractions of CNTs [19]. Therefore, the reduced interparticular interactions under low‐speed conditions can further enhance the overall separation efficiency, thereby ensuring that the supernatant is highly enriched with BNNTs while h‐BN impurities are effectively removed, establishing the low‐speed centrifugation as an optimal strategy for BNNT purification under the tested conditions.

Besides the friction (*f*) and sedimentation (*s*) coefficients, another critical factor governing the sedimentation behavior of nanotubes is the tendency of BNNTs to become entangled in a solution. Due to their high aspect ratio and strong Van der Waals interactions, BNNTs readily form bundles or entangled networks, which enhances hydrodynamic resistance and thus markedly slowing down sedimentation. For nearly spherical impurities (e.g., h‐BN), however, this phenomenon rarely occurs because they sediment more freely with minimal hydrodynamic hindrance, as supported by our calculation above. The entanglement‐induced retardation is particularly relevant in purification processes, as it facilitates the selective separation of nanotubes from spherical or short particulate impurities. Although this effect is challenging to quantify precisely, it has been consistently reported in the literatures [20, 21]. In the context of our low‐speed centrifugation system, this effect acts synergistically with the reduced centrifugal force to maintain BNNTs in the supernatant while promoting rapid impurity settling. Consequently, this synergy enhances purification efficiency without detectable structural damage to the nanotubes.

### 2.2 Experimental Verification of Low‐Speed Centrifugation Purification

**FIGURE 2:**
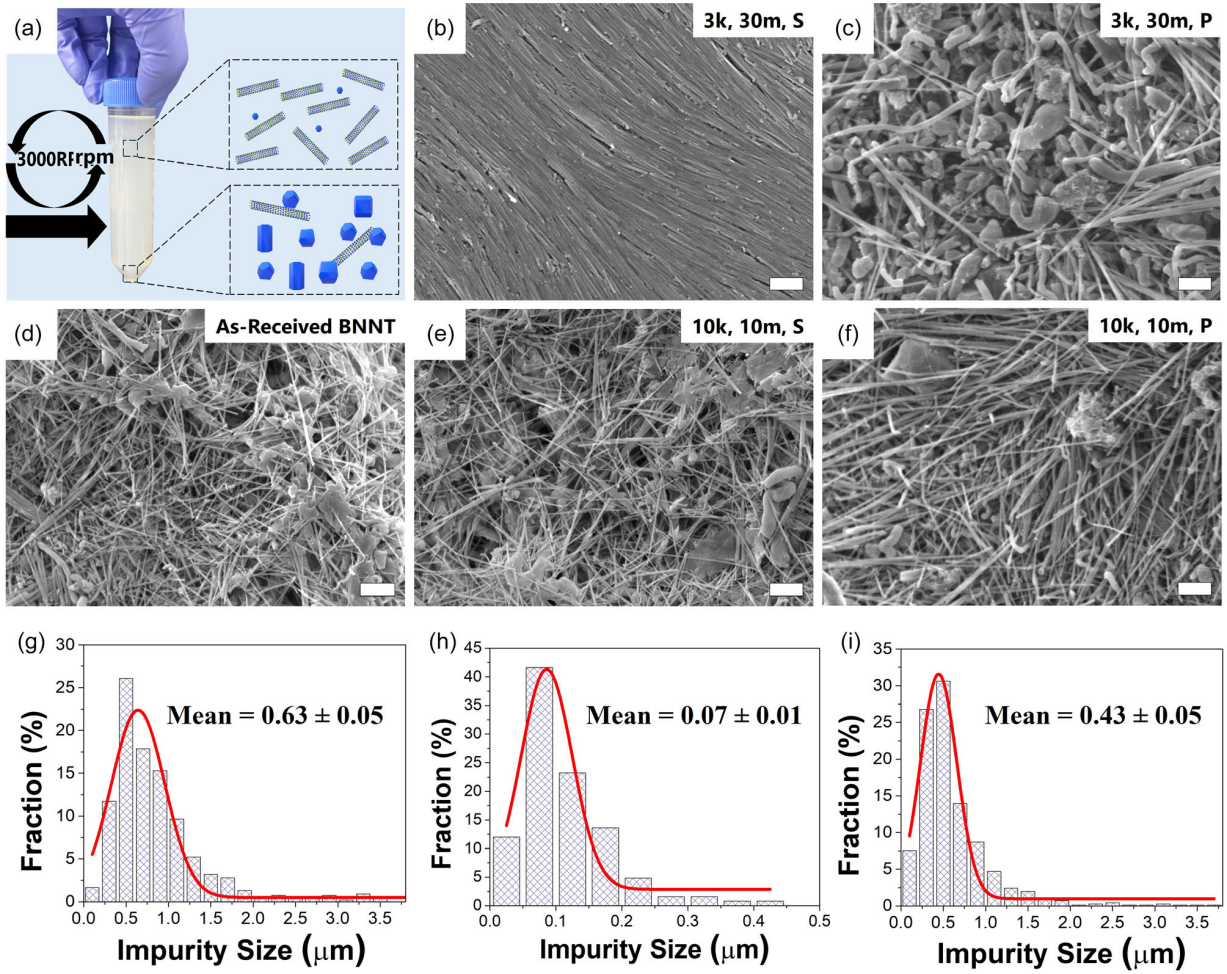
(a) Photograph of the aqueous BNNT suspension after low‐speed centrifugation (3000 rpm, 30 min) and schematics illustrating the compositions of supernatant (S) and precipitate (P). The concentrations of BNNTs and Tween 80 in the suspension are 0.1 wt% each. (b,c) SEM images of deposited films prepared from (b) the supernatant and (c) the precipitate collected after low‐speed centrifugation (3000 rpm, 30 min) of the aqueous BNNT suspension. (d) SEM image of deposited films prepared from the aqueous BNNT suspension without the centrifugation process (denoted as the as‐received BNNTs). (e,f) SEM images of deposited films prepared from (e) the supernatant and (f) the precipitate collected after high‐speed centrifugation (10,000 rpm, 10 min) of the aqueous BNNT suspension. All deposited films are prepared under identical gravity filtration conditions. Scale bars, 1 μm. (g–i) Size distributions of impurity particles measured from the deposited films prepared from (g) the as‐received BNNT and the supernatants collected after (h) low‐speed centrifugation (3000 rpm, 30 min) and (i) high‐speed centrifugation (10,000 rpm, 10 min). Each size distribution dataset is obtained by analyzing over 1000 particles from at least 10 representative SEM images; the reported uncertainties represent the standard deviation calculated from the measured impurity particle sizes.

**FIGURE 3:**
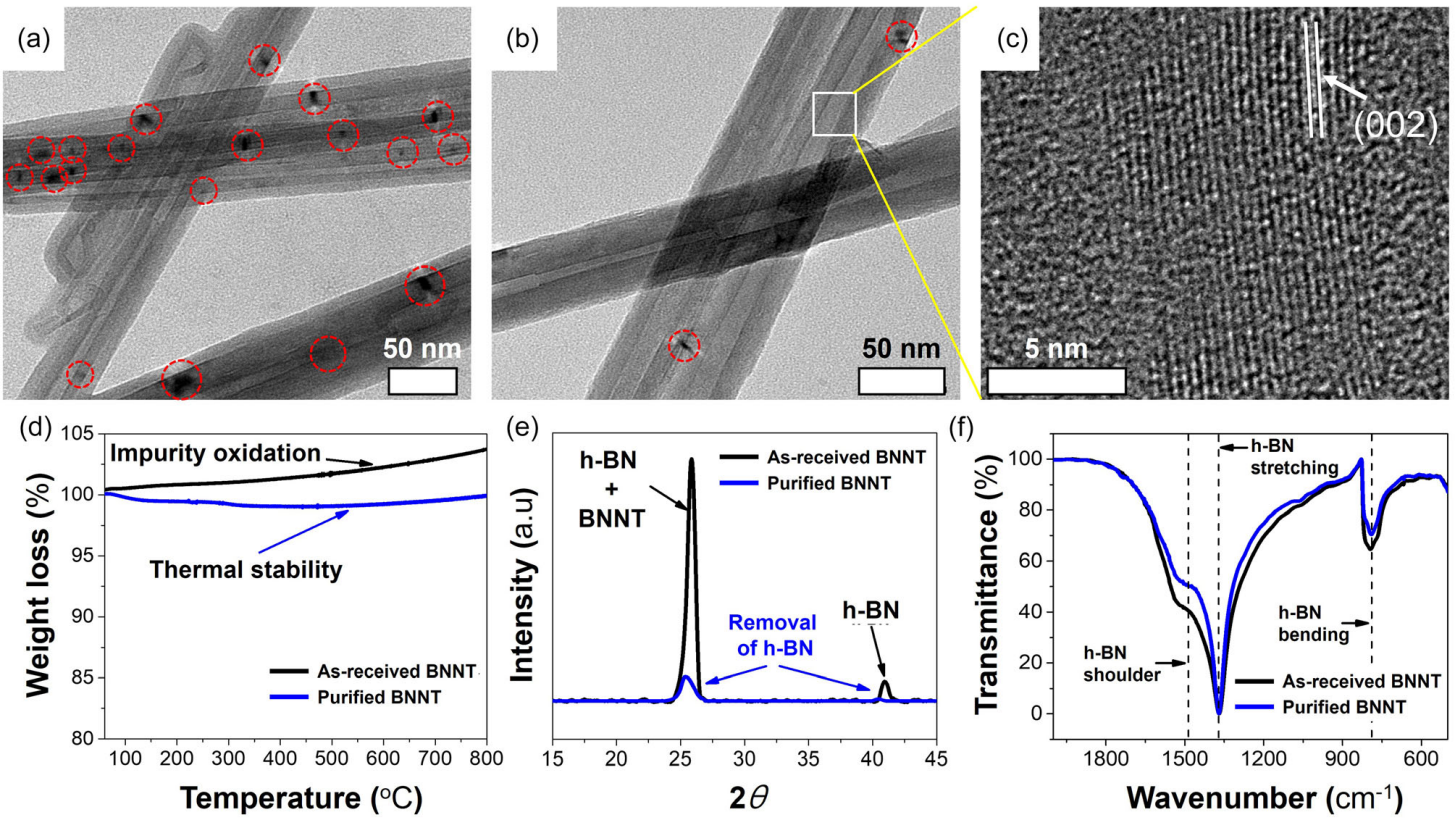
(a,b) TEM images of (a) the as‐received BNNTs and (b) the purified BNNTs via low‐speed centrifugation (3000 rpm, 30 min). Red circles indicate impurities decorated on the BNNT surfaces. Corresponding minimally processed TEM images are available in Figure S4. (c) High‐magnification TEM image of the purified BNNT. (d) Thermogravimetric analysis (TGA) curves, (e) X‐ray diffraction (XRD) patterns, and (f) Fourier‐transform infrared (FTIR) spectra of the as‐received (black lines) and purified BNNT samples (blue lines).

**FIGURE 4:**
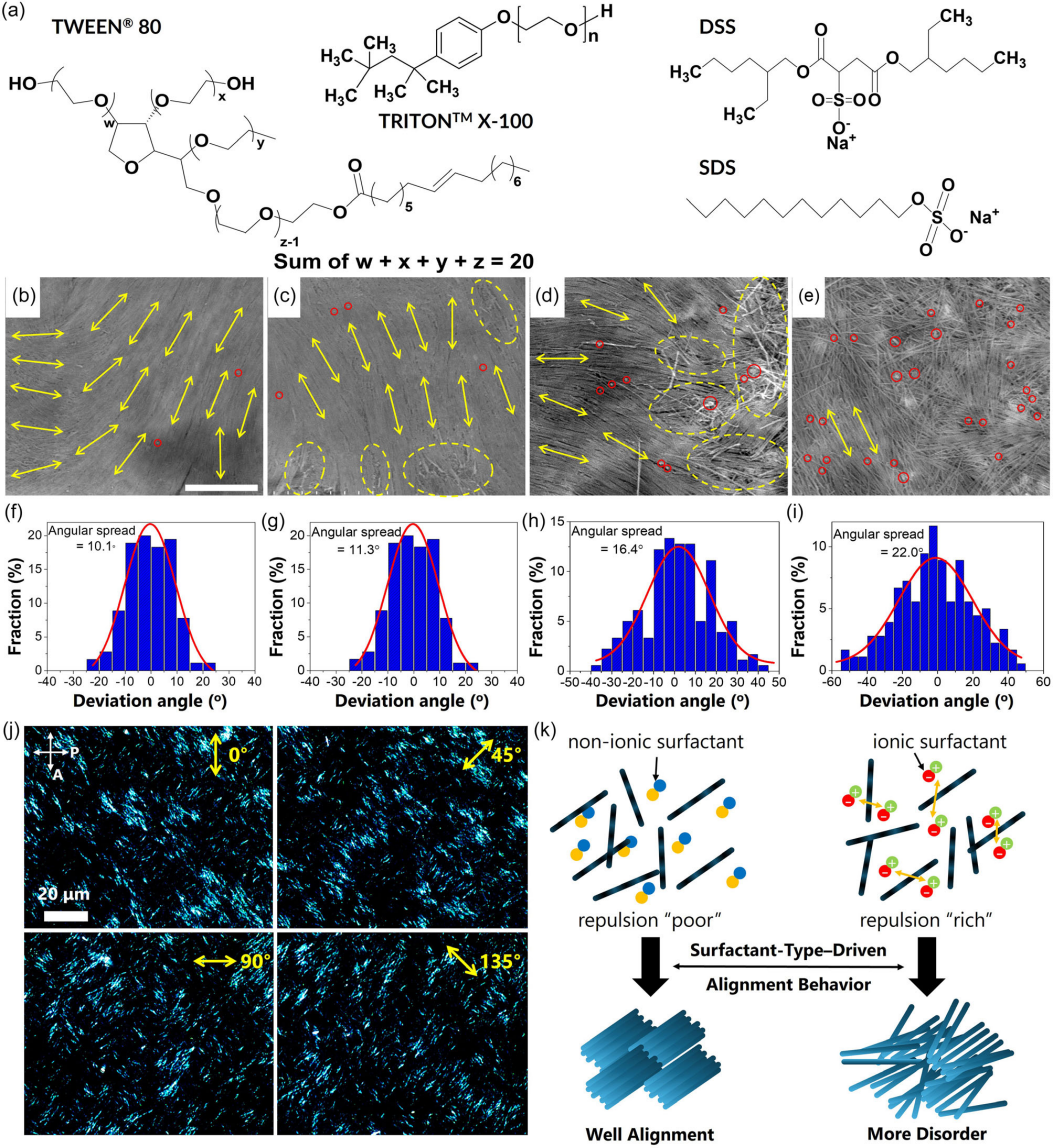
(a) Molecular structures of the surfactants used for the preparation of aqueous BNNT suspensions: nonionic Tween 80 and Triton X‐100, and anionic DSS and SDS. (b–e) SEM images of deposited BNNT films prepared from the supernatant collected after low‐speed centrifugation (3000 rpm, 30 min) of the aqueous BNNT suspensions with (b) Tween 80, (c) Triton X‐100, (d) DSS, and (e) SDS. Red circles indicate impurities. Yellow arrows indicate the orientational ordering of BNNTs. Yellow dashed circles indicate the disordered regions of BNNTs. Corresponding minimally processed SEM images are provided in Figure S4. Scale bars: 5 μm. (f–i) Angular‐distribution histograms extracted from the SEM images of the deposited BNNT films processed with (f) Tween 80, (g) Triton X‐100, (h) DSS, and (i) SDS. The angular spread is defined as the standard deviation of the angular deviation (Δ*θ*) of each BNNT bundle from the director (the dominant alignment direction). Each angular‐distribution dataset is obtained by analyzing over 180 distinct BNNT features, including individual nanotubes and nanotube bundles, from at least 3 representative SEM images. (j) Polarized optical microscopy (POM) images of the concentrated BNNT/Tween 80 dispersion (2.5 wt%) between crossed polarizers at different sample rotation angles. (k) Schematics illustrating the proposed surfactant‐regulated BNNT alignment mechanism driven by Onsager‐type excluded‐volume interactions and filtration‐assisted assembly.

To experimentally evaluate the kinetics and selectivity of the low‐speed centrifugation purification predicted by our theoretical examination, we first investigate the BNNTs using scanning electron microscopy (SEM). To this end, we centrifuge an aqueous suspension of BNNT (0.1 wt%) and Tween 80 (0.1 wt%) at 3000 rpm for 30 min and collect the precipitate and supernatant fractions (Figure 2a). The collected fractions are then filtered through a gravity filtration process that deposits solid particles onto a filter paper to form nanotube bundles as the liquid is drawn through by gravity [3, 22]. Notably, all electron microscopy analyses performed in this work (Figures 2, 3, 4 and Figures S1, S3–S5) utilize deposited BNNT films prepared with the same aqueous BNNT suspension under identical gravity filtration conditions.

In line with our theoretical predictions, we observe that the conical centrifuge tube exhibits a distinct separation between a clear supernatant and a precipitate (Figure 2a). SEM analysis reveals that the supernatant predominantly contains individualized nanotubes with minimal impurity signatures (Figure 2b), whereas the precipitate fraction consists primarily of spherical impurities and large BNNT bundles (Figure 2c). Furthermore, by analyzing aliquots collected at 0‐, 10‐, and 20‐min low‐speed centrifugations, we observe a gradual enrichment of BNNTs and a progressive reduction of impurity particles in the supernatant. Specifically, the as‐received BNNTs (0 min) show a high concentration of impurity content (amorphous boron and h‐BN platelets) along with significant aggregation (Figure 2d). After 10 min of low‐speed centrifugation, however, the supernatant already exhibits a noticeable purification effect (Figure S1a,b), which further improves after the 20‐min centrifugation (Figure S1c,d). Notably, as the centrifugation time increases, we also observe that a small fraction of shorter nanotubes begins to coprecipitate, which is consistent with our theoretical predictions regarding sedimentation kinetics.

A control study conducted at the higher centrifugal force (10,000 rpm for 10 min) yields markedly different outcomes. By analyzing SEM images, we find that both the resulting supernatant and the precipitate (Figure 2e,f) consist of a heterogeneous mixture of nanotubes and impurities, confirming that the excessive centrifugal force reduces purification selectivity by forcing BNNTs to sediment alongside non‐nanotube phases. Furthermore, we demonstrate that even with prolonged centrifugation times, the conventional high‐speed centrifugation process tends to coprecipitate both impurities and a large fraction of BNNTs (Figure S1e), thereby depleting the supernatant, which then contains only a very low concentration of smaller BNNTs. This observation is in good qualitative agreement not only with our theoretical prediction but also with a previous report [1].

The size distribution of impurity particles measured from the SEM images further highlights the impact of low‐speed centrifugation (Figure 2g–i). In the as‐received BNNT samples (Figure 2d), impurities exhibit a broad distribution ranging from the submicrometer scale to above 3.5 μm, with a dominant population in the 0.3–1 μm range (Figure 2g), reflecting the coexistence of amorphous boron clusters and h‐BN platelets. After 30 min of the low‐speed centrifugation at 3000 rpm, however, the size distribution of impurities in the supernatant narrows substantially, with the size of most remaining impurities reduced to <100 nm (Figure 2h). This corroborates that large, spherical, or platelet‐like byproducts are selectively removed, leaving the supernatant significantly enriched with individualized BNNTs (Figure 2b). In contrast, the high‐speed centrifugation condition (10,000 rpm for 10 min, Figure 2e) yields a much broader size distribution of impurities in the supernatant, with the peak remaining near 0.43 μm and a significant tail extending beyond 3 μm (Figure 2i), indicative of poor selectivity in the separation of BNNTs from impurities. Collectively, these results demonstrate that the low‐speed centrifugation provides a favorable purity–recovery balance under the tested conditions by exploiting differences in sedimentation kinetics, whereas excessive centrifugal force diminishes this balance. It is also noteworthy that the low‐speed centrifugation not only removes spherical and plate‐like impurities in a controlled manner but also avoids detectable structural damage to the BNNTs. This is reflected in the absence of broken nanotubes in the SEM analysis (further discussed in Figure 3).

As a next step, we comprehensively characterize the BNNTs to assess their structural preservation and impurity removal efficiency. Transmission electron microscopy (TEM) observations provide the first evidence of structural integrity. Both the as‐received (Figure 3a) and purified BNNTs obtained via the low‐speed centrifugation (Figure 3b) display the characteristic multiwalled tubular architecture with hollow cores. No obvious tube breakage, wall thinning, or collapse is detected, confirming that the low‐speed purification treatment does not compromise the fundamental nanotube framework. The high‐magnification TEM image of the purified BNNTs (Figure 3c) clearly shows well‐defined lattice fringes, which are periodic atomic planes corresponding to the characteristics of highly crystalline BNNTs [6]. Such lattice fringes indicate that the boron and nitrogen atoms are arranged in an ordered, defect‐free tubular structure rather than in an amorphous or disordered form. Similar lattice patterns are widely reported for as‐synthesized BNNTs in the literature, confirming that the low‐speed purification process preserves their intrinsic crystallinity [3]. Importantly, the density of high‐contrast nanoparticles decorating the nanotube surface (red circles in Figure 3a,b), which are typically attributed to catalyst residues or aggregated h‐BN platelets nucleated during synthesis [3, 23], is markedly reduced in the purified BNNTs compared to the as‐received samples. Their reduction indicates that low‐speed centrifugation effectively removes impurity particulates by sedimenting them more rapidly than the BNNTs. This observation further validates the selectivity of our method, as the heavier and more spherical impurities experience higher sedimentation velocities, leading to their successful elimination from the supernatant.

Thermogravimetric analysis (TGA) offers further compelling evidence regarding the effectiveness of low‐speed purification. Specifically, the as‐received BNNTs exhibit a distinct weight increase beginning near 300°C, which continues with increasing temperature (black line in Figure 3d). This anomalous rise is consistent with the oxidation of metallic impurities or amorphous boron phases, leading to an apparent weight gain as they form metal or boron oxides [24]. In contrast, the purified BNNTs show an essentially flat TGA curve up to 800°C (blue line in Figure 3d), demonstrating a high degree of thermal stability and the effective removal of oxidation‐prone contaminants during the purification process. In addition, the absence of significant mass variation suggests that the low‐speed purification procedure does not introduce thermally labile functional groups or structural defects into the BNNT framework [3, 7].

The structural integrity of BNNTs and the effective impurity removal after low‐speed centrifugation purification are further confirmed by X‐ray diffraction (XRD) analysis. In the as‐received BNNT sample (black line in Figure 3e), we measure two strong diffraction peaks at 2*θ* ≈ 26° and 2*θ* ≈ 41°, corresponding to the (0 0 2) and (1 0 0) lattice plane reflections of stacked h‐BN layers, respectively. Notably, the (0 0 2) peak at 2*θ* ≈ 26° contains overlapping contributions from both h‐BN and BNNTs due to their similar basal‐plane stacking distances, while the (1 0 0) peak originates primarily from the h‐BN impurities [3, 10]. After low‐speed purification (blue line in Figure 3e), we observe that the intensity of these peaks significantly diminishes, evidencing the removal of excess h‐BN. However, the (0 0 2) peak at 2*θ* ≈ 26° still remains prominent, indicating that while the h‐BN impurities are removed, the tubular architecture of the BNNTs retains its high crystallinity throughout the purification treatment. In addition, the absence of new peaks suggests that no additional crystalline phases are introduced during the purification process, aligning with the TGA results (Figure 3d).

Additionally, Fourier‐transform infrared (FTIR) spectroscopy is employed to examine the chemical structure of the BNNTs before and after low‐speed purification (Figure 3f). The measured spectra reveal two principal absorption peaks for the BNNTs (an in‐plane B–N stretching mode at 1370 cm^−1^ and an out‐of‐plane B–N bending mode at 781 cm^−1^) accompanied by a clear shoulder peak at 1487 cm^−1^. Because flat h‐BN platelets strongly enhance the stretching vibration while contributing much less to the bending mode, the prominence of this shoulder peak serves as a reliable indicator of h‐BN impurities [25]. After the purification, while the primary BNNT peaks remain almost unaltered, the transmittance of the shoulder peak noticeably increases, suggesting the removal of disordered h‐BN impurities from the BNNTs as observed in Figure 3b. We note that the observed changing trends in the FTIR signals closely align with those reported for high‐purity BNNTs in the literature [3], further validating the successful removal of h‐BN impurities by our low‐speed centrifugation method.

Furthermore, the estimated BNNT purity is evaluated by comparing the integrated FTIR peak areas in the B–N stretching region between the as‐received and purified BNNT samples (Figure 3f). Using the nominal purity of the as‐received BNNTs (∼80%) as a reference, we calculate that three independent purification batches yield estimated BNNT purities of 95%, 96%, and 93%, corresponding to an average estimated BNNT purity of 94.7% ± 1.5% (Figure S2). We note that this value is reported as an estimated BNNT purity rather than an absolute quantitative purity because FTIR intensity can be influenced by multiple factors, such as sample thickness, orientation, baseline correction, scattering, residual surfactant, and overlapping B–N vibrational modes from BNNTs and h‐BN. Therefore, to independently support this FTIR‐based purity estimation, we perform semiquantitative XRD analysis by comparing the h‐BN‐related diffraction contribution at 2*θ* = 41° with the main BN‐related diffraction peak at 2*θ* = 26° in Figure 3e; the detailed XRD peak fitting and calculation are provided in Figure S3. This analysis indicates that the normalized h‐BN‐related diffraction contribution decreases by approximately 88% after purification (yielding an estimated BNNT purity of approximately 97.6%). Because the 2*θ* = 26° peak contains contributions from both BNNTs and h‐BN, this value is used strictly as a semiquantitative indicator of h‐BN removal rather than an absolute purity measurement, further supporting the substantial removal of crystalline h‐BN impurities after low‐speed centrifugation. These purity estimates are consistent with the SEM (Figure 2), TEM (Figure 3a–c), TGA (Figure 3d), and XRD (Figure 3e) results, collectively confirming the effective enrichment of BNNTs while preserving their overall morphology. Therefore, these combined analyses robustly demonstrate that low‐speed centrifugation provides a mild and selective purification route for improving BNNT purity without causing detectable structural damage.

To evaluate the versatility of the low‐speed centrifugation strategy, we conduct a comparative test using four different aqueous BNNT suspensions containing surfactants with distinct molecular architectures that have been utilized in nanotube dispersion and purification [26] (Figure 4a): two nonionic surfactants of Tween 80 and Triton X‐100, and two ionic surfactants of dioctyl sodium sulfosuccinate (DSS) and sodium dodecyl sulfate (SDS). The concentrations of BNNTs and surfactants are fixed at 0.1 wt% each in all tested suspensions. Despite their structural differences, all four surfactants enable the efficient removal of residual contaminants, as indicated by the red circles in Figure 4b–e. In the nonionic surfactant systems (Figure 4b,c), we observe that the deposited BNNT films show smoother, denser, and more coherent BNNT networks without any large plate‐like h‐BN sheets, verifying that our low‐speed purification step effectively eliminates layered impurities while maintaining intact nanotube bundles. Similarly, the ionic surfactant systems (Figure 4d,e) also produce BNNT networks with a substantial reduction in particle‐like contaminants, confirming that the method reliably removes impurities regardless of surfactant charge. Notably, although all surfactant systems exhibit effective impurity removal, the nonionic surfactants consistently provide a more favorable purity–recovery balance compared to the ionic surfactants under the tested conditions. This is attributed to the fact that nonionic surfactants stabilize nanotube dispersions primarily through steric hindrance rather than electrostatic interactions. Such steric stabilization preserves the intrinsic hydrodynamic properties of both the BNNTs and the impurities, allowing their sedimentation behavior to remain governed by particle shape, mass, and drag. In contrast, ionic surfactants introduce strong electrostatic repulsion, which increases the effective interactions of both the BNNTs and the impurities, thus promoting cosedimentation or hindered settling. As a result, the sedimentation contrast between the high‐aspect‐ratio BNNTs and the compact impurities is reduced in the ionic surfactant systems, leading to diminished purification efficiency. In addition, high‐magnification SEM images (Figure S5) confirm that the structural integrity of BNNTs is well preserved across all surfactant environments. The resulting BNNTs retain their high‐aspect‐ratio morphology without evidence of tube fragmentation or surface damage, demonstrating that the low‐speed purification is a gentle and highly versatile process.

For a direct comparison of the estimated BNNT purity achievable by the low‐speed centrifugation across the various surfactant systems, we perform an additional targeted study with identical aqueous BNNT suspensions by centrifuging them at 10,000 rpm to evaluate the estimated purity of the BNNTs after the conventional high‐speed centrifugation (Figure S6). As shown in Figure S6a, the high‐speed centrifugation condition also produces high estimated purity values, particularly for the nonionic surfactants, with TWEEN 80 and Triton X‐100 reaching 89%–96% and 88%–94%, respectively. The estimated BNNT purity is evaluated by using the FTIR spectra of the samples, following the same method as in Figure 3f. However, the corresponding BNNT recovery is extremely low, decreasing to 2%–7% for Tween 80 and 1%–6% for Triton X‐100 depending on the centrifugation time (Figure S6b); all recovery values represent the averages of three independent gravimetric measurements with standard deviations within ±0.21%, as described in Section 4. Similar low‐recovery behavior is observed for SDS and DSS. These results indicate that high‐speed centrifugation can remove compact impurities but also promotes substantial BNNT cosedimentation, leading to a severe loss of recoverable BNNTs, which is consistent with previous research [11]. In contrast, the low‐speed centrifugation condition (3000 rpm) provides comparable estimated purity alongside a much higher recovery approaching 22% (Figure S6c), demonstrating a more favorable purity–recovery balance under the tested conditions.

Beyond impurity removal efficiency, another important observation is that the type of surfactant plays a decisive role in the self‐assembly of the BNNTs during the gravity filtration. Specifically, when the BNNT dispersions with the nonionic surfactants are filtered, we observe that a uniform alignment of BNNTs (guided by yellow arrows in Figure 4b,c) forms over the entire area of films, whereas the Triton X‐100 system exhibits limited localized regions with reduced orientational ordering (yellow circles in Figure 4c). In contrast, the deposited BNNT films processed using the ionic surfactants show significantly more extensive regions of disorder (yellow circles in Figure 4d,e). To quantitatively analyze this alignment and justify the observed ordering trend (Tween 80 > Triton X‐100 >> DSS > SDS), we perform an angular‐distribution analysis using image analysis of the SEM results in Figure 4b–e (see Section 4). In this analysis, the local orientation of the BNNT bundles is measured from the SEM images, where the dominant alignment direction is defined as the director. The measured angular deviation (Δ*θ*) of each BNNT bundle from the director is used to construct the angular distribution (Figure 4f–i), and the angular spread is defined as the standard deviation of Δ*θ*. The apparent orientational order parameter (*S*) is subsequently calculated using the equation *S* = ⟨cos(2Δ*θ*)⟩ [27]. The angular spread increases from 10.1° for Tween 80 to 11.3° for Triton X‐100, 16.4° for DSS, and 22.0° for SDS. Correspondingly, the apparent order parameter decreases in the order of Tween 80 (*S* = 0.94) > Triton X‐100 (*S* = 0.92) > DSS (*S* = 0.84) > SDS (*S* = 0.71). These quantitative values are in excellent agreement with the visual trends observed in the SEM images, confirming that nonionic surfactants promote stronger local BNNT alignment than ionic surfactants during the gravity filtration process.

The alignment behavior is further supported by polarized optical microscopy (POM) observations of the concentrated aqueous BNNT/Tween 80 dispersion (2.5 wt% each, Figure 4j). Between crossed polarizers, the dispersion exhibits clear birefringent textures that are highly dependent on the sample rotation angle. These birefringent textures and the accompanying optical anisotropy indicate the presence of local orientational ordering of BNNTs within the concentrated dispersion. Such behavior is consistent with previous reports showing that concentrated BNNT dispersions can form lyotropic liquid–crystalline phases when the nanotube concentration exceeds the threshold required for orientational ordering [3, 28]. Therefore, the narrow angular distribution and high apparent order parameter observed in the deposited BNNT film with Tween 80 (Figure 4b,f) are not simply artifacts caused by drying or membrane texture but instead originate from the transfer and fixation of preexisting anisotropic BNNT domains during gravity filtration.

Based on these findings, the surfactant‐regulated BNNT alignment can be understood as an Onsager‐type filtration‐assisted anisotropic assembly process (Figure 4k). As the dispersion becomes concentrated during solvent removal and filtration, the local BNNT concentration increases. For high‐aspect‐ratio nanotubes, this concentration increase promotes Onsager‐type excluded‐volume interactions, favoring local orientational ordering. In the nonionic surfactant systems (left in Figure 4k), especially Tween 80, steric stabilization maintains dispersion stability while allowing a sufficiently close BNNT approach for local ordering to emerge. During gravity filtration, these locally ordered, BNNT‐rich domains are further oriented, compacted, and fixed by filtration‐induced flow, capillary forces, meniscus motion, and drying, resulting in a densely packed, aligned film. In contrast, ionic surfactants generate stronger electrostatic repulsion between surfactant‐coated BNNTs (right in Figure 4k), which increases the effective interparticle distance and weakens the excluded‐volume‐driven cooperative alignment. Consequently, DSS and SDS show broader angular distributions, lower apparent order parameters, and more disordered deposited networks (Figure 4d,e,h,i). Thus, while the final film morphology cannot be assigned solely to an equilibrium Onsager nematic state, the combined SEM, angular‐distribution, and POM results robustly support a mechanism in which Onsager‐type local ordering is coupled with filtration‐assisted assembly to generate highly aligned BNNT architectures.

## 3 Conclusion

In conclusion, we demonstrate a theory‐guided and experimentally supported strategy for the gentle purification of BNNTs using low‐speed centrifugation at 3000 rpm. By using the fundamental sedimentation principles described by the Svedberg equation and Stokes’ hydrodynamic drag law, this approach takes advantage of the distinct sedimentation tendencies of compact impurities (such as amorphous boron and h‐BN platelets) versus high‐aspect‐ratio BNNTs. Under the tested conditions, compact impurities preferentially sediment, while a substantial fraction of BNNTs remains suspended, enabling selective enrichment with reduced nanotube coprecipitation. Experimental validation using SEM, TEM, XRD, and FTIR analyses supports the effective removal of nontubular impurities, yielding an estimated BNNT purity approaching 95% while preserving the overall nanotube morphology and dispersibility. Compared with the tested high‐speed centrifugation control and conventional acid‐based purification methods, this low‐speed approach offers a favorable purity–recovery balance under the tested conditions, providing a milder processing route that reduces the impurity content without obvious nanotube fragmentation or detectable structural damage.

Beyond its selectivity and mild processing conditions, the proposed purification strategy shows promising versatility across the aqueous surfactant systems examined in this study, successfully avoiding strong acid treatments while maintaining compatibility with both ionic (SDS and DSS) and nonionic (Tween 80 and Triton X‐100) surfactants. However, the purification efficiency and the deposited film morphology inherently depend on the surfactant type, with nonionic surfactants demonstrating more favorable impurity removal and local alignment behavior. Importantly, the resulting purified BNNT dispersions exhibit an enhanced tendency to form locally aligned nanotube architectures during subsequent gravity filtration. This behavior is best described as a filtration‐assisted anisotropic assembly, where Onsager‐type excluded‐volume interactions contribute alongside shear flow, capillary effects, drying kinetics, membrane effects, and anisotropic deposition. Such alignment capability may provide opportunities for anisotropic applications, including thermal management, mechanical reinforcement, and directional transport in composite and functional materials, although further in situ structural investigations are needed to fully decouple the exact thermodynamic and kinetic contributions to the alignment mechanism.

Looking forward, the combination of process simplicity, mild conditions, and sedimentation‐based selectivity suggests that low‐speed centrifugation could serve as a practical processing strategy for BNNT enrichment. Furthermore, the mechanistic framework established in this work provides a useful basis for the purification and assembly of high‐aspect‐ratio nanotubes and other one‐dimensional nanomaterials. While we successfully validate this proof‐of‐concept at the laboratory scale, we explicitly acknowledge that it is not yet a universally optimized protocol. Further studies involving more rigorous batch‐to‐batch purity and yield evaluation across larger volumes, systematic scale‐up assessments, optimization of centrifugation parameters, and in situ fundamental thermodynamic tracking of the alignment mechanism remain important for defining the broader applicability of this approach and facilitating the practical use of BNNTs in advanced material technologies.

## 4 Experimental Section/Methods

### 4.1 Material

BNNTs (∼80% purity, Naieel Technology, South Korea), Tween 80, Triton X‐100, SDS, and DSS (Sigma‐Aldrich, South Korea) were purchased and used without further purification. For all filtration processes, a PVDF filter membrane (0.22 μm, Hyundai) was utilized.

### 4.2 Characterization

The BNNT samples were characterized using a suite of advanced analytical techniques to assess their structural and chemical properties. SEM images were obtained using a JSM‐7800F PRIME field‐emission SEM equipped with Dual EDS (JEOL); the samples were prepared by filtration onto a 0.22 µm PVDF membrane (Hyundai). FTIR spectroscopy was performed using a Nicolet iS10 instrument (Thermo Fisher Scientific) to analyze chemical bonding across a wave number range of 500–4000 cm^−1^. TEM was conducted with a JEM‐2200FS (JEOL) to examine the nanoscale morphology. XRD patterns were collected using a D6 Phaser (Bruker AXS GmbH) to evaluate crystallinity. TGA was carried out using a Q‐50 thermogravimetric analyzer (TA Instruments) to assess thermal stability and composition.

### 4.3 Purification Process

BNNT dispersions were prepared by suspending the as‐received BNNTs in an aqueous solution containing surfactants. The concentrations of the BNNTs and the surfactants in the solution were fixed at 0.1 wt% each. The mixture was bath‐sonicated for 30 min to ensure uniform dispersion. Subsequently, the solution was centrifuged using a Labogene 1736R GRF‐L‐c50‐6 centrifuge at a specified speed and time, and 80% of the supernatant was collected. Gravity filtration was performed using a standard vacuum filtration setup, modified to operate without vacuum. The filter membrane was placed on a glass support, and the BNNT dispersion was introduced through a glass funnel, allowing filtration to proceed solely under gravitational force.

### 4.4 BNNT Recovery Rate Measurement

The BNNT recovery rate after centrifugation was determined using a gravimetric method. First, filter papers were dried completely and weighed to obtain the initial mass (*m*_0_). After centrifugation, a known volume of the collected supernatant was deposited onto the predried filter paper. The filter paper containing the deposited BNNT sample was then dried overnight under ambient conditions or in a drying oven until a constant mass was reached. The dried filter paper was weighed again to obtain the final mass (*m*_1_).

The recovered BNNT mass was calculated as: *m*_recovered_ = *m*_1_−*m*_0_. The BNNT recovery was calculated using: Recovery (%) = (*m*_recovered_ */m*_initial_) × 100, where *m*_initial_ is the initial mass of BNNTs used in the corresponding dispersion. Each recovery experiment was repeated three times, and the recovery values are reported as the averages of the three independent gravimetric measurements with standard deviations within ±0.21%.

### 4.5 Apparent Order Parameter Estimation (Angular‐Distribution Analysis)

The orientational ordering of BNNT bundles in the deposited films was quantified from SEM images using ImageJ software (version 1.54 g, National Institutes of Health, USA). For each surfactant system, representative SEM images were imported into ImageJ, and the local orientation angles of visible BNNT bundles were measured using the straight‐line tool. The angle values were exported and used to determine the angular deviation (Δ*θ*_i_) of each BNNT bundle from the director (the dominant alignment direction). The angular spread was defined as the standard deviation of Δ*θ*_i_, which represents the degree of orientational dispersion around the director. Subsequently, the apparent orientational order parameter (*S*) was calculated using *S* = ⟨cos(2Δ*θ*)⟩, where ⟨ ⟩ denotes averaging over all measured BNNT bundles. In this definition, *S* = 1 corresponds to perfect alignment parallel to the director, whereas lower *S* values indicate broader orientational disorder. Because the analysis was performed on dried SEM films after gravity filtration, the calculated *S* values are interpreted as apparent orientation metrics of the deposited BNNT networks rather than direct measurements of equilibrium nematic order in solution.

For the BNNT films purified using Tween 80, Triton X‐100, DSS, and SDS, the angular spreads were estimated to be 10.1°, 11.3°, 16.4°, and 22.0°, respectively. The corresponding apparent orientational order parameters were *S* = 0.94, 0.92, 0.84, and 0.71, confirming stronger local alignment in the nonionic surfactant systems compared with the ionic surfactant systems.

## Funding

This work was supported by the National Research Foundation of Korea (NRF) grant funded by the Korean government (MSIT) (RS‐2024‐00411809, RS‐2023‐00212739, RS‐2023‐00302586, 2022M3C1A3081312, RS‐2025‐11762968).

## Conflicts of Interest

The authors declare no conflicts of interest.

## Supporting information

Supplementary Material

## Data Availability

The data that support the findings of this study can be found in the Supporting Information section, including minimally processed characterization TEM and SEM data, XRD peak‐fitting information, FTIR/XRD purity‐calculation details, high‐speed centrifugation control data.
