# Supplementary material for: Theory‐Guided Low‐Speed Centrifugation for High‐Purity Boron Nitride Nanotubes
Source: Small Sci. 2026 Jul 31;6(8):e70359. doi: 10.1002/smsc.70359 (PMC13431879; doi:10.1002/smsc.70359)
Supplement: Supplementary file 1 — Supplementary Material [file SMSC-6-e70359-s001.pdf]

Supporting Information

Theory-Guided Low-Speed Centrifugation for High-Purity Boron Nitride Nanotubes

*Thang Quoc Huynh, Jun Hyung Im, Eunsu Cho, Jin-Kang Choi, Kwang-Suk Oh, Young-Ki Kim**

Department of Chemical Engineering, Pohang University of Science and Technology (POSTECH), Pohang 37673, Republic of Korea.

Correspondence to: Prof. Young-Ki Kim, email: ykkim@postech.ac.kr

Part 1

Sedimentation and Frictional Coefficient Calculations for BNNT and h-BN Impurity

1. Introduction

This part provides detailed calculations for the sedimentation coefficient (s), frictional coefficient (*f*), and sedimentation speeds (*v*) at 10,000 rpm and 3,000 rpm for a spherical h-BN impurity (radius 100nm) and four types of BNNTs. The BNNTs utilized in this analysis consist of four representative types: i) BNNT-1 (length *L* = 5 μm x diameter *D* = 5 nm), ii) BNNT-2 (5 μm x 50 nm), iii) BNNT-3 (10 μm x 5 nm), and iv) BNNT-4 (10 μm x 50 nm). The calculations use the Svedberg equation and Stokes' law for the spherical impurity, with the Broersma/Tirado approximation for cylindrical BNNT.

2 General Equations and Assumptions

2.1 Svedberg Equation

The sedimentation coefficient (*s*) is given by :

$$s = m\,(1 - \bar{v}\,\rho_0)/f$$

where:

m : particle mass (kg),

$\bar{v} = 1/\rho_p$: partial specific volume of the particle (m^3/kg),

ρ_p : density of the particle (kg/m^3)

ρ_0 : density of the solvent (kg/m^3),

f : frictional coefficient (kg/s).

Sedimentation coefficient is reported in seconds (s) and Svedberg units (S = 10^{-13} s).

2.2 Sedimentation Speed

The sedimentation speed (v) is

$$v = s\cdot\omega^2\cdot r_r$$

where:

- $\omega = 2\pi\cdot(\text{RPM}/60)$: angular speed (rad/s)
- r_r : rotor radius (m).

2.3 Frictional Coefficient

For the spherical h-BN impurity:

$$f = 6\pi\cdot\eta\cdot r_p$$

For cylindrical BNNT (Broersma/Tirado approximation):

$$f = 3\pi\cdot\eta\cdot L/(\ln(2L/D) - 0.307)$$

Where:

- η : medium viscosity (Pa·s),
- r_p : radius for spherical particle (m),
- L : length of cylinder (m),
- D : diameter of cylinder (m).

2.4 Mass

For spherical h-BN:

$$m = (4/3)\pi \cdot r^3 \cdot \rho_p$$

For cylindrical BNNT:

$$m = \pi \cdot (D/2)^2 \cdot L \cdot \rho_p$$

2.5 Assumptions and Constants

- Medium : water at 25°C, η = 8.9×10^{-4} kg/(m·s), ρ_0 = 1000kg/m^3.
- Particle density : ρ_p = 2100 kg/m^3, so $\bar{v}$ = 1/ρ_p = 1/2100 ≈ 4.762 ×10^{-4}m^3/kg.
- Buoyancy factor : 1 – $\rho_0 \cdot \bar{v}$ = 1 – (1000/2100) = 0.524
- Rotor radius : r_r = 0.0985m.
- Angular velocities :

  - RPM = 10,000 : ω =2π·(10000/60) ≈ 1047.198rad/s, ω^2 ≈ 1.096×10^{6}rad^2/s^2.

  - RPM = 3,000 : ω =2π·(3000/60) ≈ 314.159rad/s, ω^2 ≈ 9.870×10^{4}rad^2/s^2.

3 Calculations

**Dimensions**	***f* (kg/s)**	***s* (S)**	***v* (µm/s) at 3,000 rpm**	***v* (µm/s) at 10,000 rpm**
**h-BN** (r_p = 100nm)	1.678×10^{-9}	2.747×10^{4}	26.69	296.6
**BNNT-4** ($L \times D$ = 10μm × 50nm)	1.476×10^{-8}	1.464×10^{4}	14.22	158.0
**BNNT-3** ($L \times D$ = 10μm × 5nm)	1.050×10^{-8}	2.057×10^{2}	0.200	2.221
**BNNT-2** ($L \times D$ = 5μm × 50nm)	8.403×10^{-9}	1.285×10^{4}	12.49	138.7
**BNNT-1** ($L \times D$ = 5μm × 5nm)	5.750×10^{-9}	1.878×10^{2}	0.1825	2.027

Part 2

Supporting Figure

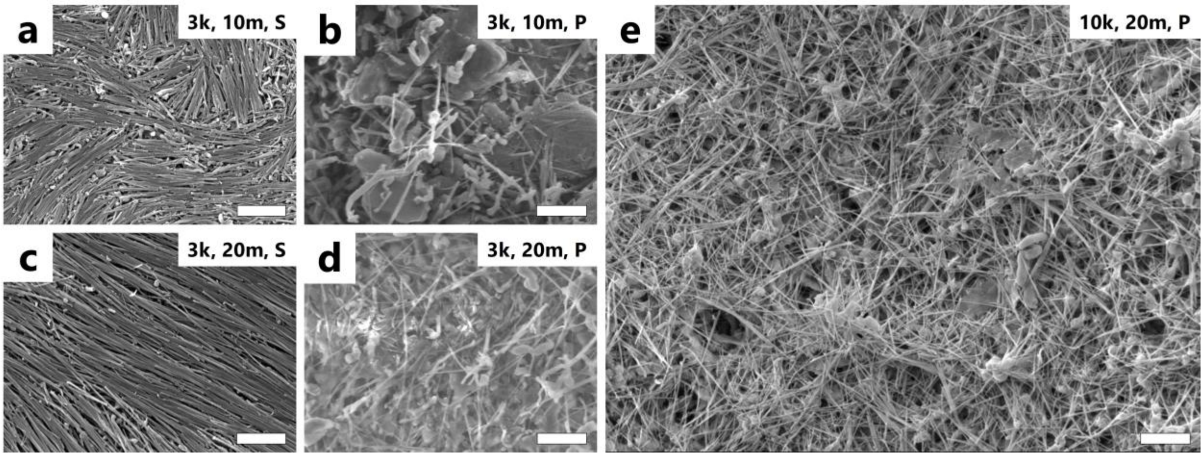

Figure S1. SEM images of BNNT samples processed using TWEEN 80 surfactant. (a, c) The supernatant and (b, d) the precipitate collected after the low-speed centrifugation at 3,000 rpm for (a ,b) 10 and (c, d) 20 min, showing the effective and selective purification of BNNTs. With increasing centrifugation time, the purity of the recovered BNNTs enhances, while a small fraction of shorter nanotubes begins to co-precipitate, consistent with theoretical predictions. (e) Precipitate obtained after the high-speed centrifugation at 10,000 rpm for 20 min. A significant amount of BNNTs co-sediments together with residual impurities, demonstrating the low purification selectivity of the high-speed centrifugation approach. Scale bars, 2μm.

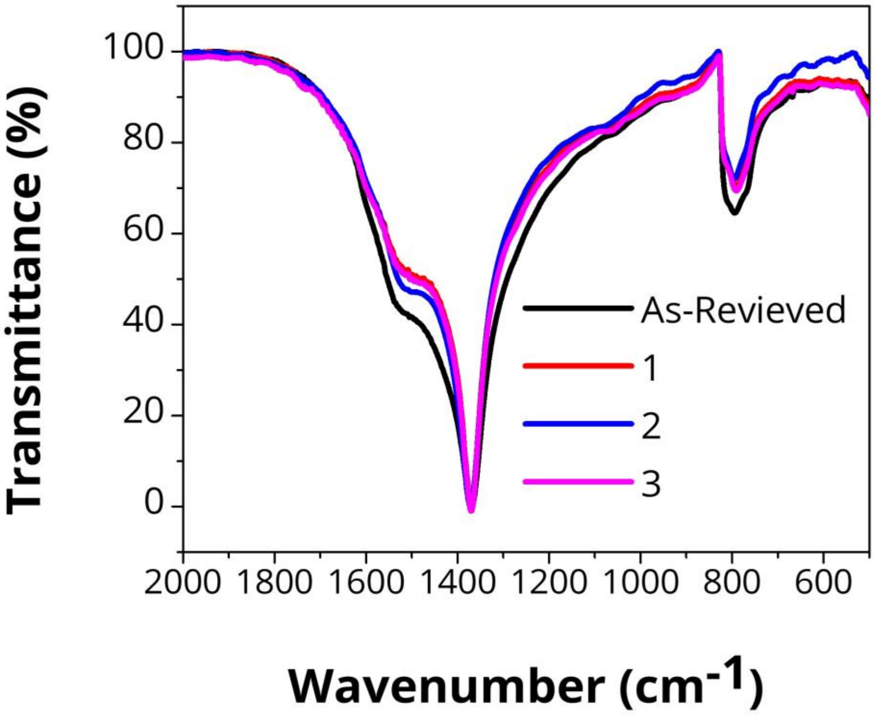

Figure S2. FTIR analysis of three independent purification batches. FTIR spectra are collected from three independently purified BNNT batches, denoted as Batches 1, 2, and 3. The FTIR-based purity estimates are 95%, 96%, and 93%, respectively, corresponding to an average estimated purity of 94.7 ± 1.5%.

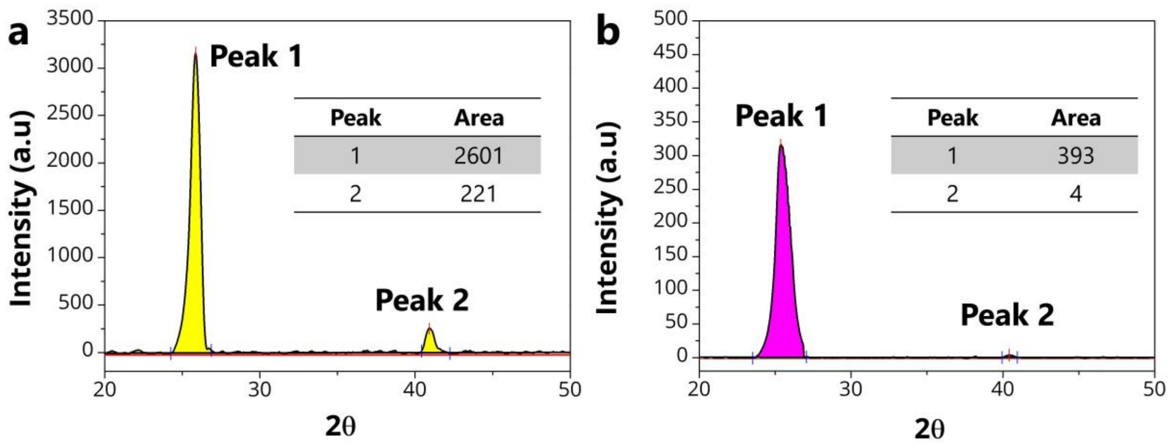

Figure S3. XRD peak-area analysis for estimating h-BN impurity removal. (a, b) Fitted XRD patterns of (a) the as-received BNNTs and (b) the purified BNNTs. The peak-area ratios are calculated by fitting the XRD peaks using Origin 9 software. The h-BN-related diffraction contribution around $2\theta = 41°$ is normalized to the main BN-related peak around $2\theta = 26°$ using $A_{41°}/A_{26°}$. This ratio is used as a semi-quantitative indicator of crystalline h-BN removal after purification.

Semi-quantitative XRD analysis of h-BN impurity removal : To provide an independent indicator of crystalline h-BN impurity removal, the XRD patterns of the as-received and purified BNNT samples are analyzed using a normalized peak-area ratio. Because absolute XRD intensity can be influenced by sample loading, packing density, preferred orientation, crystallinity, and background correction, the absolute intensity of the h-BN-related peak is not used directly. Instead, the integrated area of the h-BN-related diffraction contribution around $2\theta = 41°$ is normalized to the main BN-related diffraction peak around $2\theta = 26°$:

$$R_{\mathrm{hBN}} = \frac{A_{41°}}{A_{26°}}$$

where $A_{41°}$ is the integrated area of the h-BN-related diffraction contribution and $A_{26°}$ is the integrated area of the main BN-related diffraction peak. This ratio was used as a relative h-BN impurity index rather than an absolute BNNT purity value.

For the as-received BNNT sample (**Figure S3 a**):

$$R_{\text{hBN,raw}} = (221/2601) = 0.0850$$

For the purified BNNT sample (**Figure S3 b**):

$$R_{\text{hBN,purified}} = (4/393) = 0.0102$$

Reduction of h-BN-related contribution:

$$\text{Reduction (\%)} = [1 - (R_{\text{hBN,purified}} / R_{\text{hBN,raw}})]\cdot 100 = [1 - (0.0102/0.0850)]\cdot 100 = 88.0\%$$

Therefore, the normalized h-BN-related XRD contribution decreased by approximately 88.0% after low-speed centrifugation. This result supports the substantial removal of crystalline h-BN impurities after purification.

Assuming that the impurity fraction in the as-received BNNT sample is mainly h-BN and that the nominal purity of the as-received BNNTs is approximately 80%, the initial h-BN impurity fraction can be estimated as 20%. Based on the 88.0% reduction in the normalized h-BN-related XRD contribution, the remaining h-BN fraction after purification is estimated as 20%·(1−0.88) = 2.4%. Thus, the XRD-supported estimated BNNT purity can be approximated as 97.6%. We emphasize that this value should not be interpreted as an absolute purity because the XRD peak-area ratio is affected by crystallinity, preferred orientation, packing, and peak overlap. Instead, it provides semi-quantitative support for the removal of crystalline h-BN impurities. The XRD result is consistent with the FTIR-based purity estimation and the SEM, TEM, and TGA analyses.

In addition, the FTIR-based purity estimation is performed for three independent purification batches. The estimated purities were 95%, 96%, and 93%, giving an average value of 94.7% and standard deviation of 1.5%. Therefore, the estimated BNNT purity is reported as: 94.7 ±1.5%.

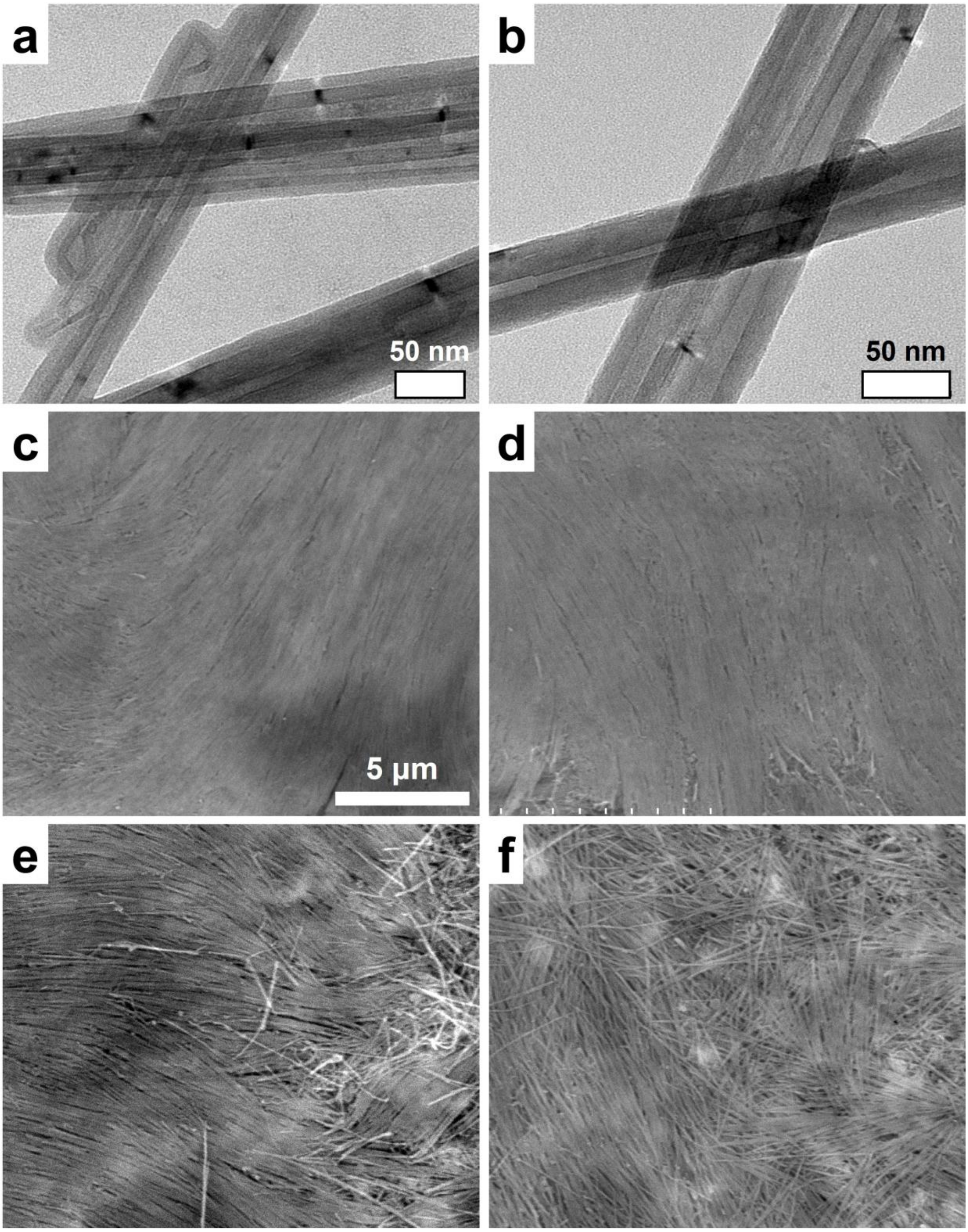

Figure S4. (a, b) Minimally processed TEM images of the as-received and purified BNNTs utilized in (a) Figure 3a and (b) Figure 3b, respectively. (c-f) Minimally processed SEM images of the deposited BNNT films utilized in (c) Figure 4b, (d) Figure 4c, (e) Figure 4d, and (f) Figure 4e, respectively.

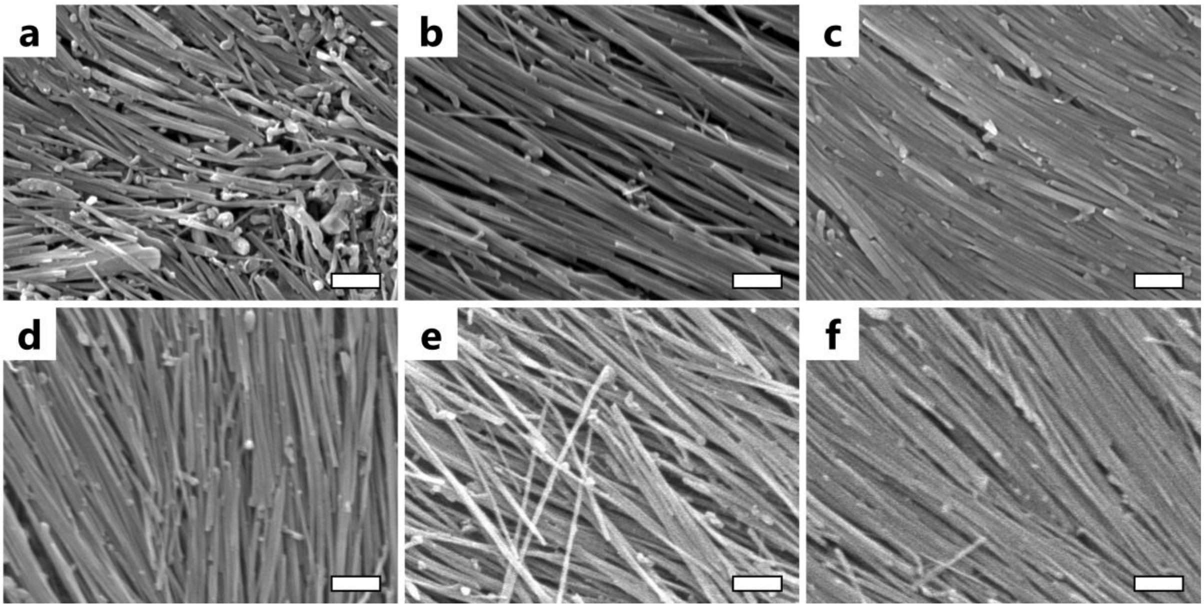

Figure S5. High-magnification SEM images of the purified BNNTs processed with different surfactants. (a–c) BNNTs collected after the low-speed centrifugation at 3,000 rpm for (a) 10, (b) 20, and (c) 30 min using TWEEN 80. As the centrifugation time increases, the samples show progressively reduced impurity content and a high apparent order parameter. (d–f) BNNTs purified after the low-speed centrifugation at 3,000 rpm for 30 min using (d) SDS, (e) DSS, and (f) Triton X-100. These samples also exhibit reduced impurity contents with well-preserved nanotube structures, demonstrating that our purification strategy is compatible with a broad range of surfactant systems. Scale bars, 500 nm.

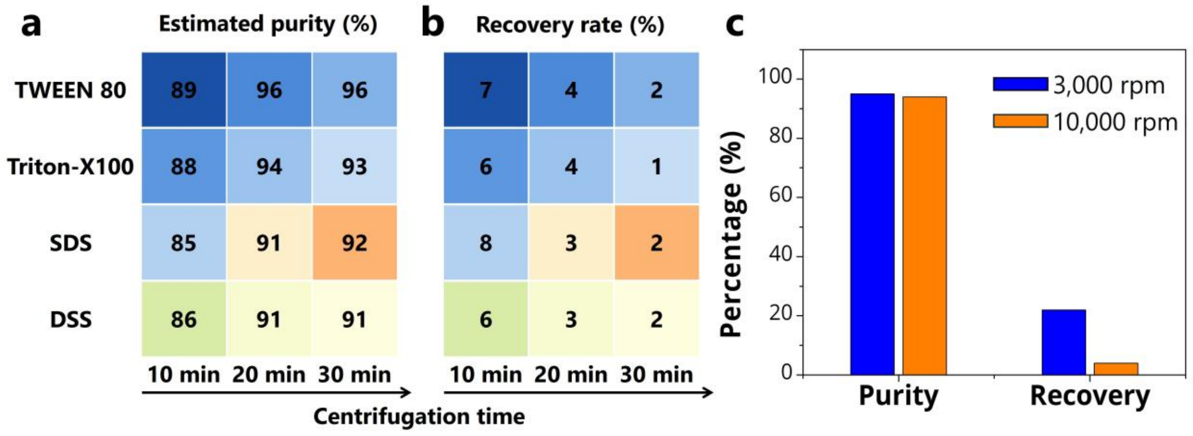

Figure S6. (a) The estimated purity and (b) recovery rate of BNNTs after high-speed centrifugation (10,000 rpm) of the aqueous BNNT suspensions with respect to centrifugation times (10, 20, and 30 min) and surfactant types (TWEEN 80, Triton X-100, SDS, and DSS). The concentrations of BNNTs and surfactants are fixed at 0.1 wt% each, and the upper 80% of the supernatant fraction is collected for analysis. All recovery values in (b) represent the averages of the three independent gravimetric measurements with standard deviations within ±0.21%, as described in the Methods of the main text. (c) Comparison plot for the maximum estimated purities and recovery rates of BNNTs after high- and low-speed centrifugations under the tested conditions in (a, b).
